# Widespread epistasis regulates glucose homeostasis and gene expression

**DOI:** 10.1371/journal.pgen.1007025

**Published:** 2017-09-29

**Authors:** Anlu Chen, Yang Liu, Scott M. Williams, Nathan Morris, David A. Buchner

**Affiliations:** 1 Department of Biochemistry, Case Western Reserve University, Cleveland, OH, United States of America; 2 Department of Population and Quantitative Health Sciences, Case Western Reserve University, Cleveland, OH, United States of America; 3 Department of Genetics and Genome Sciences, Case Western Reserve University, Cleveland, OH, United States of America; Stanford University School of Medicine, UNITED STATES

## Abstract

The relative contributions of additive versus non-additive interactions in the regulation of complex traits remains controversial. This may be in part because large-scale epistasis has traditionally been difficult to detect in complex, multi-cellular organisms. We hypothesized that it would be easier to detect interactions using mouse chromosome substitution strains that simultaneously incorporate allelic variation in many genes on a controlled genetic background. Analyzing metabolic traits and gene expression levels in the offspring of a series of crosses between mouse chromosome substitution strains demonstrated that inter-chromosomal epistasis was a dominant feature of these complex traits. Epistasis typically accounted for a larger proportion of the heritable effects than those due solely to additive effects. These epistatic interactions typically resulted in trait values returning to the levels of the parental CSS host strain. Due to the large epistatic effects, analyses that did not account for interactions consistently underestimated the true effect sizes due to allelic variation or failed to detect the loci controlling trait variation. These studies demonstrate that epistatic interactions are a common feature of complex traits and thus identifying these interactions is key to understanding their genetic regulation.

## Author summary

Most complex traits and diseases are regulated by the combined influence of multiple genetic variants. However, it remains controversial whether these genetic variants independently influence complex traits, and therefore the impact of each variant could be simply added together (additivity), or whether the variants work together to influence trait variation, in which case the combined impact of multiple variants would differ from the summed impact of each individual variant (epistasis). In this study in mice, we discovered that the genetic regulation of blood sugar levels and gene expression in the liver were predominantly controlled by non-additive interactions, whereas body weight was predominantly controlled by additive interactions. Remarkably, the expression level of nearly 25% of all genes in the liver was controlled by non-additive interactions. The non-additive interactions typically acted to return trait values to the levels detected in control mice, thus contributing to a reduction in trait variation. We also demonstrated that not accounting for non-additive interactions significantly underestimated the phenotypic effect of a genetic variant on a particular genetic background, suggesting that many previously identified risk loci may have significantly larger effects on disease susceptibility in a subset of individuals. These studies highlight the importance of understanding interactions between genetic variants to better understand disease risk and personalize clinical care.

## Introduction

The genetic basis of complex traits and diseases results from the combined action of many genetic variants [1]. However, it remains unclear whether these variants act individually in an additive manner or via non-additive epistatic interactions. Epistasis has been widely observed in model organisms such as *S*. *cerevisiae* [2,3], *C*. *elegans* [4], *D*. *melanogaster* [5] and *M*. *musculus* [6]. However, it has been more difficult to detect in humans, potentially due to their diverse genetic backgrounds, low allele frequencies, limited sample sizes, complexity of interactions, insufficient effect sizes, and methodological limitations [7,8]. Nonetheless, a number of genome-wide interaction-based association studies in humans have provided evidence for epistasis in a variety of complex traits and diseases [9–15]. However, concerns remain over whether observed epistatic interactions are due to statistical or experimental artifacts [16,17].

To better understand the contribution of epistasis to complex traits, we studied mouse chromosome substitution strains (CSSs) [18]. For each CSS, a single chromosome in a host strain is replaced by the corresponding chromosome from a donor strain. This provides an efficient model for mapping quantitative trait loci (QTLs) on a fixed genetic background. This is in contrast to populations with many segregating variants such as advanced intercross lines [19], heterogeneous stocks [20], or typical analyses in humans. Given the putative importance of genetic background effects in complex traits [21,22], we hypothesized the fixed genetic backgrounds of CSSs can provide a novel means for detecting genetic interactions on a large-scale [18,23]. Previous studies of CSSs with only a single substituted chromosome suggested that non-additive epistatic interactions between loci were a dominant feature of complex traits [6]. However, to identify the interacting loci, or at least their chromosomal locations, requires the analysis of genetic variation in multiple genomic contexts [24]. We thus extended the analysis of single chromosome substitutions by analyzing a series of CSSs with either one or two substituted chromosomes, collectively representing the pairwise interactions between genetic variants on the substituted chromosomes. This experimental design can directly identify and map loci that are regulated by epistasis by analyzing the phenotypic effects of genetic variants on multiple fixed genetic backgrounds. Here we report the widespread effects of epistasis in controlling complex traits and gene expression. The detection of true epistatic interactions will improve our understanding of trait heritability and genetic architecture as well as provide insights into the biological pathways that underlie disease pathophysiology [25]. Knowing about epistasis will also be essential for guiding precision medicine-based decisions by interpreting specific variants in appropriate contexts.

## Results

### Contribution of epistasis to metabolic traits

Body weight and fasting plasma glucose levels were measured in a total of 766 control and CSS mice (S1 and S2 Tables, S1 Fig). The CSSs included 240 mice that were heterozygous for one A/J-derived chromosome and 444 mice that were heterozygous for two different A/J-derived chromosomes, both on otherwise B6 backgrounds. The CSSs with two A/J-derived chromosomes represented all pairwise interactions between the individual A/J-derived chromosomes. For example, comparisons were made between strain B6, strains (B6.A3 x B6)F1 and (B6 x B6.A10)F1 which were both heterozygous for a single A/J-derived chromosome (Chr. 3 and 10, respectively), and strain (B6.A3 x B6.A10)F1 which was heterozygous for A/J-derived chromosomes 3 and 10 (S2 Fig). A complete list of the strains analyzed is shown in S2 Table. Quantitative trait loci (QTLs) were identified for both body weight and plasma glucose levels that were due to main effects and interaction effects. Of note, due to the nature of the CSS experimental design, the regions defined by the identified QTLs correspond to the entire substituted chromosome and contain many allelic variants that may contribute to trait regulation. Additionally, due to the study design, only QTLs with dominant or semi-dominant effects could be assessed.

Joint F-tests for main effects on body weight indicated that the chromosome substitutions influenced body weight (males p = 0.0028; females p = 0.0008; meta p = 1.4e-05). Similarly, joint F-tests tests for main effects on plasma glucose levels demonstrated a significant effect of the chromosome substitutions (males p = 0.0082; females p = 0.00011; meta p = 1.4e-05). QTLs with main effects on body weight were mapped to chromosomes 8 (main effect: 1.23g; average effect: 1.02g) and 17 (main effect: -1.13g; average effect: -1.11g) (S3 Table). Note that we define main effects as the effect of a chromosome substitution as estimated by a model which includes all pairwise interaction terms, thus taking into account context-dependent genetic background effects. In contrast, the average effect is estimated using a model that does not include any interaction terms; the latter is similar to the analyses performed in a typical GWAS study. QTLs with main effects on fasting glucose were mapped to chromosomes 3 (main effect: 25.0 mg/dL; average effect: 9.61 mg/dL), 5 (main effect: 15.6 mg/dL; average effect: 6.02 mg/dL), and 4 (main effect: 17.5 mg/dL; average effect: 6.61 mg/dL) (S3 Table).

Joint F-tests for interaction effects on body weight were not significant (males p = 0.19; females p = 0.83; meta p = 0.44), and therefore epistatic interactions on body weight were not further investigated. However, joint F-tests for interaction effects on plasma glucose demonstrated the importance of epistasis in regulating this trait (males p = 0.002; females p = 0.003; meta p = 8.99e-05). In fact, among the males and females respectively, epistasis accounted for 43% (95% confidence interval: 23%-75%) and 72% (95% confidence interval: 37%-97%) of the heritable effects on plasma glucose levels. The discrepant results for the contribution of interactions to body weight and plasma glucose are likely reflected in the difference between whether QTLs for these traits were detected using the main effect model or the average effect model (S3 Table). For plasma glucose, only 1 of the 3 QTLs identified using the main effect model was also identified using the average effect model, and no new QTLs were identified with the average effect model. In contrast, both of the QTLs for body weight identified using the main effect model were also identified using the average effect model, and 2 new QTLs were identified on chromosomes 6 and 10. This suggests that for a trait regulated by epistatic interactions, the ability to successfully identify QTLs is greatly enhanced by accounting for these interactions. However, for a trait regulated primarily by additive effects, a model incorporating interactions can be detrimental to QTL identification.

**Fig 1:**
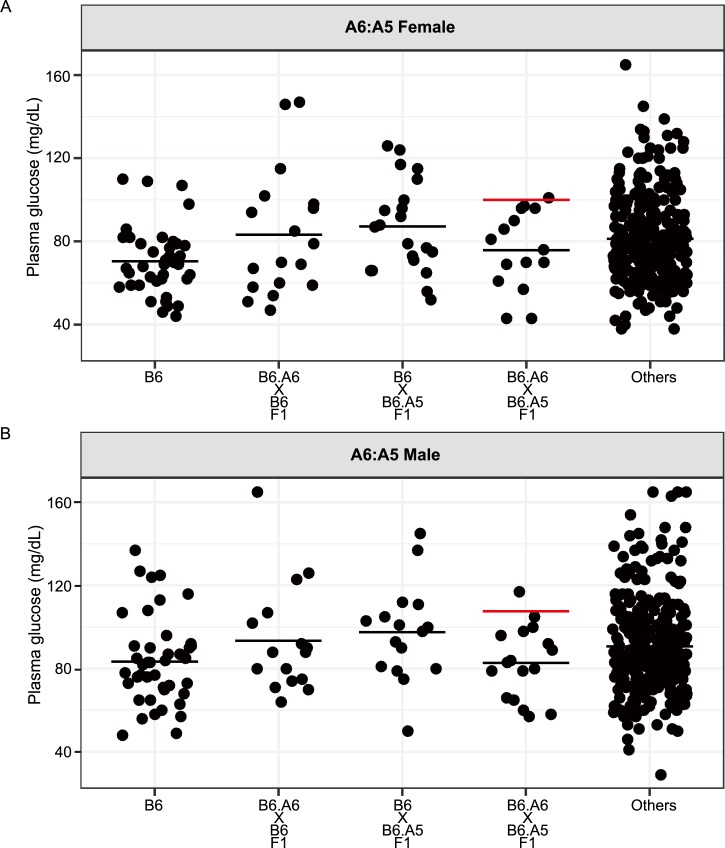
Inter-chromosomal epistasis between chromosomes 5 and 6 regulates fasting plasma glucose levels in mice. Plasma glucose levels were measured in 5-week-old (A) female and (B) male mice that were fasted overnight. Each dot represents the glucose level of a single mouse. “Others” represents the data from all mice in this study excluding the other 4 strains shown in that panel. The black horizontal line indicates the mean glucose level for each group. The red horizontal line indicates the predicted trait level based on a model of additivity.

To identify specific epistatic interactions, we tested explicit hypotheses for inter-chromosomal pairwise interactions on plasma glucose levels. Among the 15 CSS crosses analyzed, 5 crosses demonstrated inter-chromosomal epistatic interactions that altered plasma glucose levels (Fig 1, S3 and S4 Figs). Interestingly, in all 5 crosses demonstrating interactions, one chromosome substitution increased fasting glucose levels relative to the control B6 strain. These main effects raised plasma glucose levels by an average of 12.3 mg/dL in males and 17.8 mg/dL in females. However, in all 5 observed interactions the average plasma glucose levels in the double CSSs were closer to the control B6 strain than any single CSS was. Furthermore, in 4 of the 5 interactions, the plasma glucose levels in the double CSS did not differ statistically from the control strain B6 (p value > 0.1). Thus, the chromosome substitution driving the increase in plasma glucose on a B6 background had no effect on glucose levels when the genetic background was altered by the second chromosome substitution.

### Regulation of gene expression by epistasis

**Fig 2:**
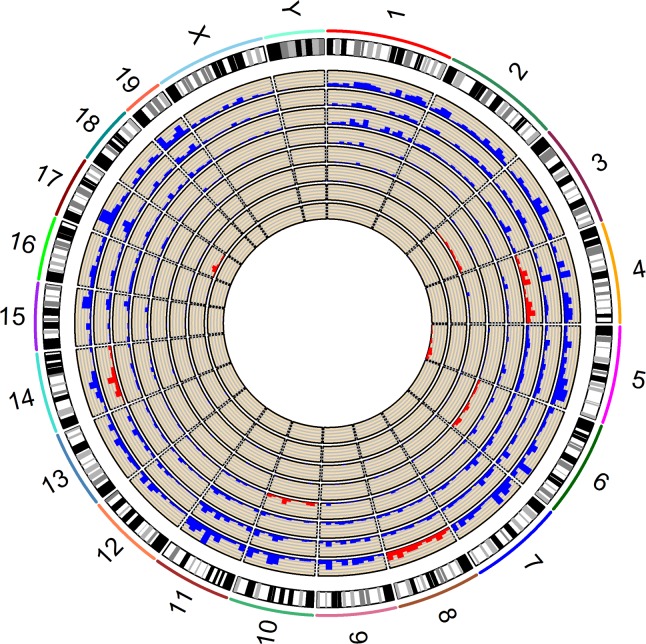
Identification of meQTLs that regulate hepatic gene expression. A circos plot of meQTL locations in the genome where each layer of the circle represents the comparison between a CSS strain and control B6 mice. From the inner circle, the CSS strains are (B6 x B6.A5)F1, (B6.17 x B6)F1, (B6.A3 x B6)F1, (B6.A6 x B6)F1, (B6 x B6.A10)F1, (B6 x B6.A4)F1, (B6.A14 x B6)F1 and (B6 x B6.A8)F1. Cis-meQTLs and trans-meQTLs are marked with red and blue, respectively. The width of each chromosome is proportional to its physical size. The height of each meQTL bar is proportional to the number of meQTLs in that genomic interval.

As hepatic gluconeogenesis is a key determinant of plasma glucose levels in healthy insulin-sensitive mice [26], the hepatic gene expression patterns of control and CSS male mice were analyzed to better understand the molecular mechanisms underlying the epistatic regulation of plasma glucose. The RNA-Seq data was filtered for genes expressed in the liver, leaving 13,289 genes that were tested for differential expression associated with both main and interaction effects. A total of 6,101 main effect expression QTLs (meQTLs) were identified (FDR < 0.05) (Fig 2, S4 Table). Those meQTL genes located on the substituted chromosome were classified as cis-meQTLs (Fig 2, red) whereas the meQTL genes not located on the substituted chromosome were classified as trans-meQTLs (Fig 2, blue). Among all possible genes regulated by a cis-meQTL, on average 11.48% of these genes in each strain had a cis-meQTL (range: 5.54% - 22.09%) (S5 Table). Similarly, among all possible genes regulated by a trans-meQTL, on average 5.42% (range: 0.08% to 19.26%) of these genes were regulated by a trans-meQTL (S5 Table). The percentage of cis- and trans-meQTLs in each strain demonstrated a strong positive correlation (Spearman’s r = 1.0) but the proportion of cis-eQTLs was always greater than the proportion of trans-eQTLs. Strain (B6 x B6.A8)F1 had both the highest percentage of genes with cis-meQTLs (22.09%) and trans-meQTLs (19.26%), whereas strain (B6 x B6.A5)F1 had both the lowest percentage of genes with cis-meQTLs (5.54%) and trans-meQTLs (0.08%). This suggests that trans-meQTLs are being driven by the cumulative action of many cis-effects rather than a single or small number or major transcriptional regulators (S5 Fig). Among the genes regulated by a meQTL(s), 41.98% (1615 out of 3847) were regulated by multiple meQTLs (Range: 2–6) (S6 and S7 Tables). For example, *Brca2* is regulated by 5 trans-meQTLs mapped to chromosomes 4, 6, 8, 10 and 14 (S6 Fig, S7 Table), demonstrating that hepatic *Brca2* expression is regulated by allelic variation throughout the genome. In addition to the well-known role of *Brca2* in breast cancer susceptibility, *Brca2* has been implicated in hepatocellular carcinoma risk [27–29].

In addition to the meQTLs regulated by substitution of a single chromosome, the analysis of double CSSs enabled the detection of eQTLs with additive and interaction effects between the substituted chromosomes. The expression of *Zkscan3* represents an example of additivity, with the substitution of A/J-derived chromosomes 8 and 17 each individually increasing the expression of *Zkscan3* relative to control B6 mice (S7A Fig). In the double CSS strain (B6.A17 x B6.A8)F1, the effects of each individual chromosome substitution are combined in an additive manner to result in yet higher expression than either of the single CSSs (S7A Fig). The additive effects of the *Zkscan3* meQTLs detected by RNA-Seq were confirmed by quantitative reverse transcription PCR (S7B Fig), as were 4/5 additional meQTLs demonstrating additivity (S8 Table).

**Fig 3:**
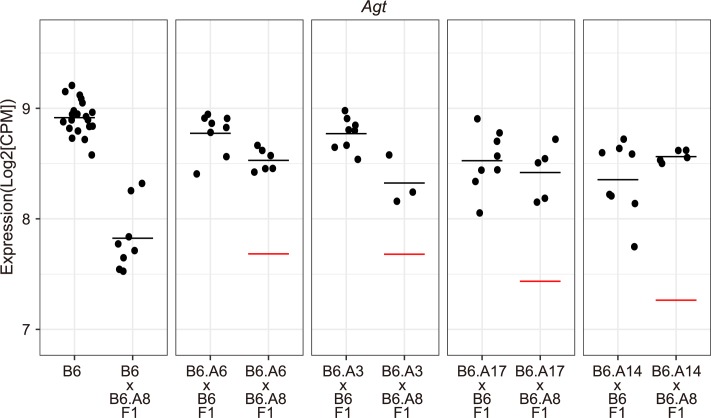
Identification of 4 ieQTLs that regulate the hepatic expression of Agt. Gene expression levels of Agt in the liver are shown for strain B6, 5 single CSS strains, and 4 double CSS strains. Each dot represents Agt expression in an individual mouse. The mean value for each strain is indicated by a solid line. The expected expression level of Agt in the double CSS strains based on a model of additivity is indicated with a red line. The Agt gene is located on mouse chromosome 8.

In addition to examples of additivity, interaction expression QTLs (ieQTLs) were identified that were jointly regulated by genetic variation on two substituted chromosomes. The ieQTLs, similar to the meQTLs, were divided into cis-ieQTLS and trans-ieQTLs, with cis-ieQTLs defined by differentially expressed genes located on either one of the two substituted chromosomes and trans-ieQTLs representing differentially expressed genes that are not located on either substituted chromosome. A total of 4,283 ieQTLs were identified (S9 Table). Among all possible genes regulated by a cis-ieQTL or trans-ieQTL, 2.01% and 2.16% of genes were regulated by a cis- or trans-ieQTL respectively (Table 1). The combination of A/J-derived chromosomes 8 and 14 yielded the most ieQTLs (n = 2,305) including cis-ieQTLs regulating the expression of 17.56% of all genes on chromosomes 8 or 14 and trans-ieQTLs regulating the expression of 17.32% of all genes throughout the remainder of the genome. Overall, the ieQTLs demonstrated a similar positive correlation as the meQTLs (Spearman’s r = 0.92) (S8 Fig), although there was no enrichment for cis-ieQTLs. Among the genes regulated by an ieQTL(s), 32.35% (945 out of 2921) were regulated by multiple ieQTLs (Range: 2–7) (S10 and S11 Tables). For example, *Agt* expression is decreased in strain (B6.A8 x B6)F1 relative to control B6 mice; however, interactions between one or more alleles on chromosome 8 and chromosomes 6, 3, 17, and 14 all result in expression levels of *Agt* that did not differ from the control strain (Fig 3).

**Table 1:** Interection effects on gene expression.

Cross	Cis-ieQTLs			Trans-ieQTLs			Subtypes of ieQTLs			
	Percentage of genes with cis-ieQTL	Number of cis-ieQTLs	Number of genes on substituted chromosomes	Percentage of genes with trans-ieQTL	Number of trans-ieQTLs	Number of genes on other chromosomes	Percentage of genes with synergistic ieQTLs	Number of synergistic ieQTLs	Percentage of genes with antagonistic ieQTLs	Number of antagonistic ieQTLs
A14:A8	17.56%	199	1133	17.32%	2106	12156	6%	129	94%	2176
A6:A8	6.81%	96	1409	5.89%	700	11880	2%	15	98%	781
A14:A4	3.86%	48	1244	3.57%	430	12045	6%	31	94%	447
A6:A10	1.81%	24	1325	0.58%	69	11964	1%	1	99%	92
A14:A10	1.62%	17	1049	2.05%	251	12240	1%	3	99%	265
A6:A4	0.86%	13	1520	0.58%	68	11769	0%	0	100%	81
A3:A8	0.77%	11	1434	0.77%	91	11855	2%	2	98%	100
A3:A10	0.44%	6	1350	0.56%	67	11939	0%	0	100%	73
A17:A8	0.23%	3	1329	0.00%	0	11960	0%	0	100%	3
A17:A4	0.14%	2	1440	0.48%	57	11849	2%	1	98%	58
A17:A10	0.08%	1	1245	0.03%	4	12044	0%	0	100%	5
A14:A5	0.00%	0	1410	0.17%	20	11879	0%	0	0%	0
A17:A5	0.00%	0	1606	0.00%	0	11683	0%	0	0%	0
A3:A5	0.00%	0	1711	0.00%	0	11578	0%	0	0%	0
A6:A5	0.00%	0	1686	0.00%	0	11603	0%	0	100%	20
All	2.01%	420	20891	2.16%	3863	178444	4%	182	96%	4101

### Context-dependent effects on gene expression

**Fig 4:**
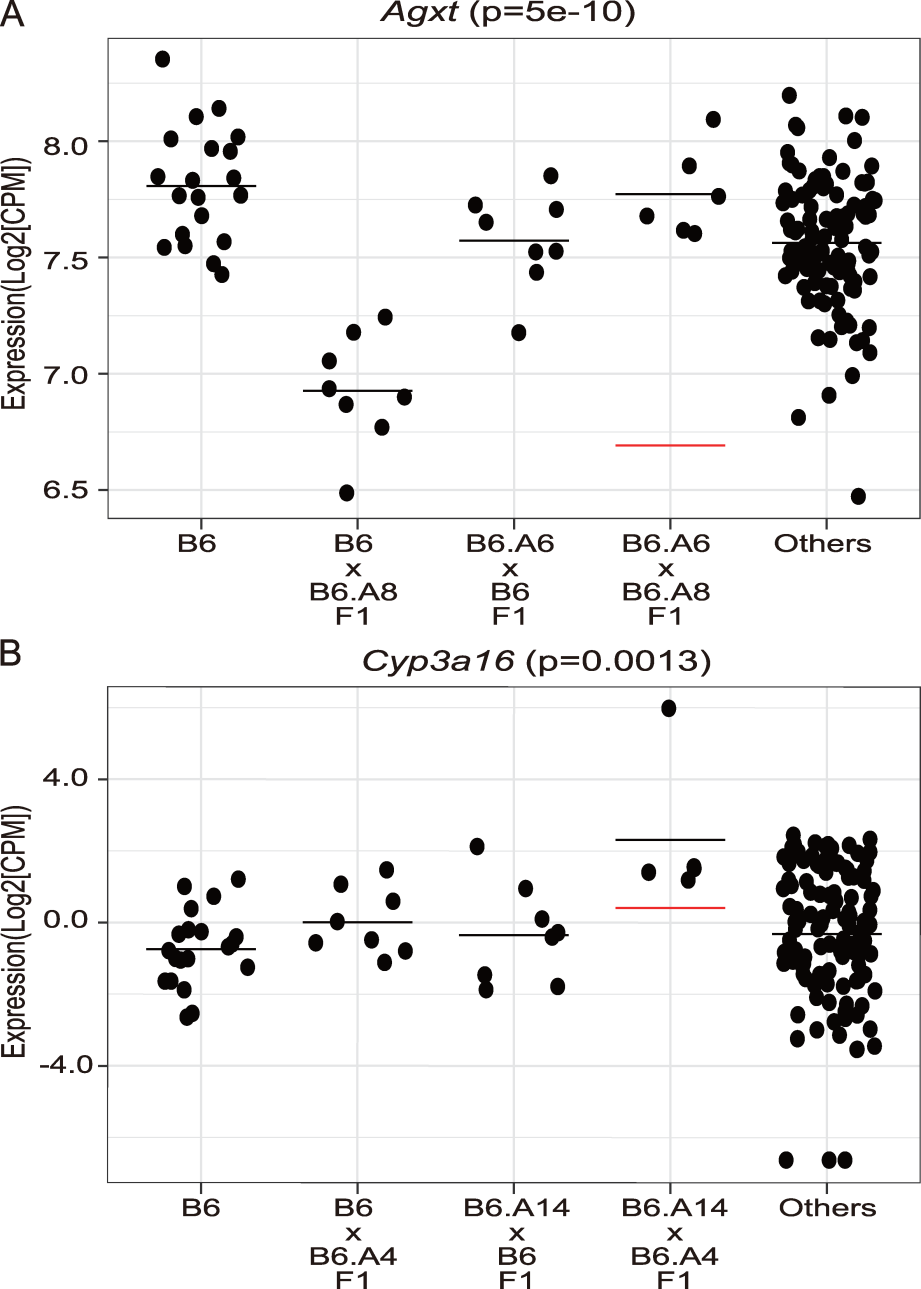
Examples of synergistic and antagonistic ieQTLs. Each dot represents the gene expression data from one mouse. The horizontal bar indicates the mean value for each strain (A) An antagonistic ieQTL regulates the expression of *Agxt* in the liver. (B) A synergistic ieQTL regulates the expression of *Cyp3a16* in the liver. The red horizontal line indicates the predicted trait level based on a model of additivity.

We next tested whether the interaction effects on gene expression were synergistic (positive epistasis) or antagonistic (negative epistasis) (S9 Fig). Synergistic refers to an increased difference in gene expression levels between the double CSS and the control B6 strain beyond that expected based on an additive model, whereas antagonistic refers to a decreased difference. The regulation of *Agxt* was an example of an antagonistic interaction, with main effects from substituted chromosomes 6 and 8 each individually decreasing *Agxt* expression, whereas this effect was lost in the double chromosome substitution strain (Fig 4A). In contrast, the regulation of *Cyp3a16* represented an example of synergistic interaction with the detection of an ieQTL in the absence of a meQTLs (Fig 4B). Among the ieQTLs, antagonistic interactions accounted for 96% (n = 4101) while synergistic interactions accounted for 4% (n = 182) (Table 1). Remarkably, for 80% of the antagonistic interactions (3285/4101), gene expression in one or both of the single CSSs differed from the control B6 strain (a meQTL), whereas expression in the double CSS reverted to control levels (p > 0.1 relative to strain B6). To again validate the RNA-Seq data using an independent method, RT-qPCR was performed for a subset of genes with antagonistic (n = 13) and synergistic (n = 10) interactions. Replication by RT-qPCR confirmed the detection of epistasis in 61% (p <0.05) of the genes tested (Antagonistic: 8/13; Synergistic: 6/10) (S8 Table).

### Significant contribution of epistasis to trait heritability

**Fig 5:**
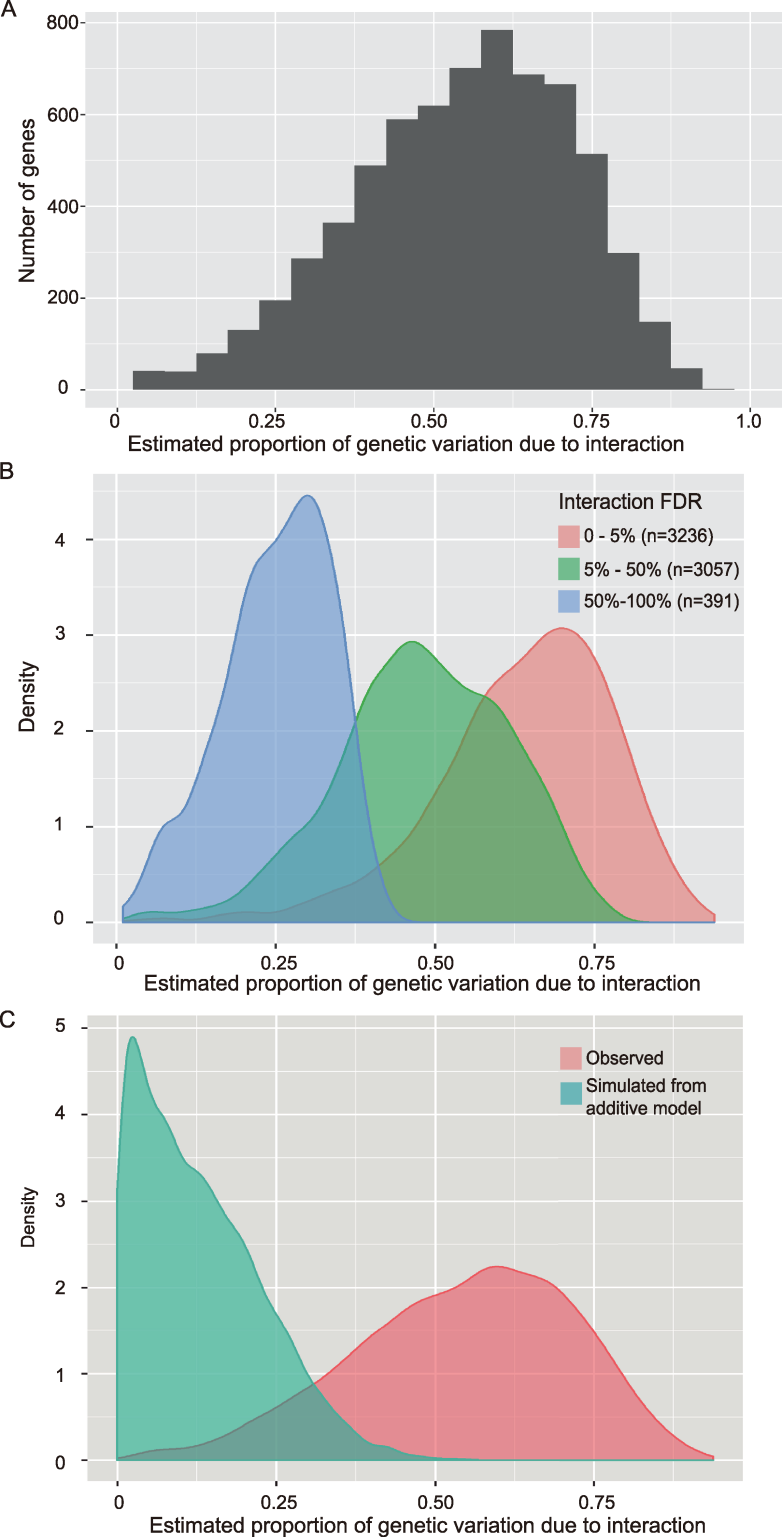
Contribution of epistasis to the genetic regulation of hepatic gene expression. Diagrams representing the estimated proportion of genetic variation due to interactions for (A) all genes expressed in the mouse liver whose expression was under genetic control in the CSS strains studied, (B) the same data segregated based on the statistical evidence supporting an effect of interaction on gene expression, and (C) a comparison of the genes with the most significant evidence for regulation by genetic interactions (FDR < 0.05) and a simulation study with artificial data that model the absence of any genetic interactions.

Given that the ieQTLs regulated approximately 2% of all genes expressed in the liver (Table 1), we sought to quantify the contribution of genetic interactions to the heritable component of all genes. First, an empirical Bayes quasi-likelihood F-test identified 6,684 genes out of the 12,325 genes expressed in the liver for which there was evidence of genetic control within the population of CSSs (FDR<0.05). The average proportion of heritable variation attributable to interactions across these genes was 0.56 (1^st^ quartile: 0.43 – 3^rd^ quartile: 0.68) (Fig 5A). When the same analysis was restricted to only genes with a statistically significant (FDR<0.05) contribution of interactions to gene expression levels (n = 3,236 genes), the proportion of heritable variation attributable to interactions increased to 0.66 (1^st^ quartile: 0.56, 3^rd^ quartile: 0.74) (Fig 5B). For comparison, a simulation study was conducted using artificial data to model pure additivity in the absence of interactions, with a resulting estimate of heritability of 0.13 (1^st^ quartile: 0.05, 3^rd^ quartile: 0.19) (Fig 5C), which provides an estimate of the background noise in this measurement. Thus, genetic interactions are a major contributor to the regulation of gene expression.

## Discussion

CSSs, which have a simplified and fixed genetic background, were used to identify widespread and likely concurrent epistatic interactions. This systematic analysis of mammalian double CSSs demonstrated that epistatic interactions controlled the majority of the heritable variation in both fasting plasma glucose levels and hepatic gene expression (Fig 5). Among genes expressed in the liver, the expression level of 24% were regulated, at least in part, by epistasis (Fig 5). This number is remarkable considering that only dominant or semi-dominant effects were tested, only a single tissue and time point were examined, allelic variation from only two inbred strains of mice were included, and only 15 pairwise strain combinations of CSSs were tested out of a possible 462 combinations of double CSSs. The prevalence of epistatic interactions provides a potential molecular mechanism underlying the highly dependent nature of complex traits on genetic background [21,22,30,31]. Interpreting the effect of individual allelic variants will thus be severely limited by population-style analyses that fail to account for possible contextual effects. Nonetheless, progress is being made in this field, including in diseases such as multiple sclerosis (MS), which is a complex genetic disease whose risk is highly associated with family history [32]. For example, MS risk alleles in DDX39B (rs2523506) and IL7R (rs2523506A) together significantly increase MS risk considerably more than either variant independently [15]. Based on the considerable number of interactions detected in the CSS crosses, context-dependent interactions such as that between DDX39B and IL7R in MS are likely widespread and may therefore represent a significant source of missing heritability for complex traits and diseases [33,34].

Although epistasis was a dominant factor regulating fasting glucose levels, the same effect was not detected in the regulation of body weight. It is not clear if this is due to different genetic architectures between these two traits or whether this was due to the limited genetic variation between the B6 and A/J strains. The body weight studies were conducted in mice fed a standard rodent chow, whereas differences in body weight between strains B6 and A/J are significantly more pronounced when challenged with a high-fat diet [35,36]. Alternately, a recent meta-analysis of trait heritability in twin studies identified significant variation in the role of additive and non-additive variation among different traits, with suggestive evidence for non-additive effects in 31% of traits [37]. Among the traits analyzed, genetic regulation of neurological, cardiovascular, and ophthalmological traits were among the most consistent with solely additive effects, whereas traits related to reproduction and dermatology were more often consistent with non-additive interactions. Among the metabolic traits studied, 40% of the 464 traits studied were consistent with a contribution of non-additive interactions [37]. It is interesting to speculate whether some traits that may have a more direct effect on fitness (e.g. reproduction) are more likely to involve multiple non-additive effectors in order to maintain a narrow phenotypic or developmental range [38].

Although many inter-chromosomal non-additive interactions were identified in mice, it remains unclear whether these interactions are attributable to bigenic gene-gene interactions or to higher-order epistasis involving multiple loci located on a substituted chromosome. Studies in yeast that dissected the genetic architecture of epistasis demonstrated that gene-gene interactions played a minor role among the heritable effects attributable to epistasis, thus primarily implicating higher order interactions [2]. Yet, other studies in yeast that methodically tested pairs of gene knockouts for interactions identified a number of gene-gene interactions [39]. Additional evidence for both high-order epistasis with three, four, and even more mutations [40] as well as bigenic gene-gene interactions [41] have been identified, and it seems likely that both will underlie interactions detected in the CSS studies. This is because the use of CSSs to study the allelic variation found on an entire chromosome in tandem equally enables the detection of bigenic and higher-order interactions, although it does not distinguish between these two possibilities by identifying the number of contributing variants on the substituted chromosomes without further mapping studies. This property of CSSs may contribute to the robust detection of epistasis using the CSS experimental platform relative to genetic mapping studies in populations with many independently segregating variants, which are often underpowered to identify higher-order interactions [42]. However, to formally test this and determine the relative contribution of each, higher resolution genetic mapping of the epistatic interactions will be necessary to better understand their molecular nature [43]. Higher resolution mapping studies should eventually shed light on whether the chromosome-level properties discovered in this study are consistent with those for SNP-level interactions. Based on previous studies of complex trait QTLs in single-CSS studies, chromosome-level QTLs demonstrated a similar genetic architecture as that found in higher resolution QTLs including large effect sizes, similar direction of effects, and suggestive evidence of widespread epistasis [23,44]. Thus, it seems likely that discoveries made based on chromosome-level analysis of epistasis, will apply equally to studies involving individual genetic variants. For example, genetic variants in *Cntnap2* were identified by higher resolution mapping studies of chromosome-level QTLs in CSSs, that were associated with opposing effects on body weight depending on epistatic interactions with intra-chromosomal variation in the genetic background [45].

Perhaps the most significant outcome of the epistasis detected was the high degree of constancy in the light of context dependence, such that the interactions usually returned trait values to the levels detected in control mice. Remarkably, this is just as Waddington predicted 75 years ago, a phenomenon he referred to as canalization [46] and has been observed in previous studies[47–51]. Canalization refers to the likelihood of an organism to proceed towards one developmental outcome, despite variation in the process along the way. This variation can be influenced by among other things the numerous functional genetic variants present in a typical human genome, which may contain thousands of variants that alter gene function [52]. We find that the overwhelming majority of genetic interactions return trait values to levels seen in control strains, which would act to reduce phenotypic variation among developmental outcomes. Studies of epistasis in tomato plants detected by analyzing short chromosomal regions on different genetic backgrounds identified a similar bias towards antagonistic epistasis relative to synergistic epistasis[50]. A bias towards antagonistic interactions was also detected in large-scale gene-gene interactions studies in yeast, although with a lower frequency of antagonistic relative to synergistic interactions[49,53]. Thus, our results are concordant with other studies that the majority of epistatic interactions are antagonistic, and together suggest that when larger tracts of DNA are assessed for interactions the effects are even more likely to be antagonistic. This robustness in the face of considerable genetic variation is central to the underlying properties of canalization. These genetic interactions therefore represent a mechanism for storing genetic variation within a population, without reducing individual fitness. This stored genetic variation could then enable populations to more quickly adapt to environmental changes [54].

Finally, the consistently greater effect sizes of main effects relative to average effects suggests that GWAS-type studies, in both human and model organisms, consistently underestimate true effect sizes in at least a subset of individuals. For example, a large F2 intercross between inbred mice carrying a mutation that results in a nonfunctional allele of the growth hormone releasing hormone receptor (*Ghrhr*) on either a B6 or C3H genetic background identified widespread antagonistic epistasis, albeit with small contributions to overall trait heritability relative to additive effects [47]. Similarly, epistatic interactions were identified in the Diversity Outbred mice resulting in small contributions to the overall heritability of metabolic-related traits [55]. These studies contrast the large contribution of epistasis to trait heritability identified using the CSS paradigm (Fig 5), mirroring the contrasting portraits of genetic architecture identified based on differing genetic structures of these experimental populations [23]. The CSS paradigm examines context-dependent effects on individual genotypes and typically identifies QTLs with large effect sizes. Alternatively, GWAS-type studies average effects across a population of heterogeneous genotypes and typically identify QTLs with small phenotypic effects. However, perhaps most relevant is that the relatively simpler genotypes of CSSs enable greater depth analyzing fewer unique genotypes, potentially capturing what would be rare genotypic combinations in a segregating cross or human population. Therefore, the key to enabling precision medicine, which like the CSS studies is focused on the effect of a variant on one specific genetic background, is to identify in which subset of individuals a particular variant has a significant effect. The consideration of epistasis in treatment, although in its infancy, remains a promising avenue for improving clinical treatment regimens, including predicting drug response in tumors [56] and guiding antibiotic drug-resistance [57]. However, true precision medicine will necessitate a more comprehensive understanding of how genetic background, across many loci, affects single variant substitutions.

## Materials and methods

### Ethics statement

All mice were cared for as described under the Guide for the Care and Use of Animals, eighth edition (2011) and all experiments were approved by IACUC and carried out in an AAALAC approved facility. The IACUC protocol numbers were 2013–0098 and 2016–0064. Mice were anesthetized with isoflurane prior to retro-orbital bleeding and subsequently euthanized by cervical dislocation for tissue collection.

### Mice

Chromosome substitution strains (CSS) and control strains were purchased from The Jackson Laboratory. These strains include C57BL/6J-Chr3^A/J^/NaJ mice (Stock #004381) (B6.A3), C57BL/6J-Chr4^A/J^/NaJ mice (Stock #004382) (B6.A4), C57BL/6J-Chr5^A/J^/NaJ mice (Stock #004383) (B6.A5), C57BL/6J-Chr6^A/J^/NaJ mice (Stock #004384) (B6.A6), C57BL/6J-Chr8^A/J^/NaJ mice (Stock #004386) (B6.A8), C57BL/6J-Chr10^A/J^/NaJ mice (Stock #004388) (B6.A10), C57BL/6J-Chr14^A/J^/NaJ mice (Stock #004392) (B6.A14), C57BL/6J-Chr17^A/J^/NaJ mice (Stock #004395) (B6.A17) and C57BL/6J (Stock #000664). Mice were maintained by brother-sister matings. All mice used for experiments were obtained from breeder colonies at Case Western Reserve University. Mice were housed in ventilated racks with access to food and water *ad libitum* and maintained at 21°C on a 12-hour light/12-hour dark cycle. Male mice from strains B6, B6.A4, B6.A5, B6.A10 strains and B6.A8 were bred with female mice from strains B6, B6.A3, B6.A6, B6.A14 and B6.A17 strain. The offspring were weaned at 3 weeks of age. The number of offspring analyzed from each cross is shown in S2 Table for both body weight and plasma glucose, although glucose levels were not measured in one mouse each from the following strains: (B6 x B6.A10)F1, (B6.A14 x B6)F1, (B6.A17 x B6.A10)F1, (B6.A3 x B6.A10)F1, (B6.A6 x B6.A4)F1, (B6.A14 x B6.A5)F1 and (B6.A6 x B6.A5)F1. The mice analyzed from each cross were derived from at least three independent breeding cages. No blinding to the genotypes was undertaken.

### Mouse phenotyping

At 5 weeks of age, mice were fasted 16 hours overnight and body weight was measured. Mice were anesthetized with isoflurane and fasting blood glucose levels were measured via retro-orbital bleeds using an OneTouch Ultra2 meter (LifeScan, Milpitas, CA, USA). Mice were subsequently euthanized by cervical dislocation and the caudate lobe of the liver was collected and immediately placed in RNAlater (Thermo Fisher Scientific, Waltham, MA, USA).

### Trait analysis

To analyze the body weight and fasting plasma glucose data, linear regression was used with a main effects term and a term for each pairwise interaction for the males and females separately. In the glucose data, 5 observations were Winserized by setting a ceiling of 4 median absolute deviations from the median. Any values larger than the ceiling (165 mg/dL) were set to the ceiling. Additionally, interactions where one of the crosses contained less than 5 mice were not analyzed leading to the removal of the (B6.A4 x B6.A3)F1 mice, the female (B6.A8 x B6.A14)F1 and the male (B6.A8 x B6.A3)F1 mice. For each trait and for each sex, we estimated a linear model with the following predictors: (1) maternal substitution, (2) paternal substitution and (3) the interaction of maternal by paternal substitution. In these models, the reference strain was B6. The sexes may potentially differ in residual variance and in the effect of the chromosome substitutions (i.e. gene by sex interaction). To handle these differences transparently, we estimated and reported models for each sex separately. Within each of the above models, two joint linear hypothesis tests were performed of the following hypothesis: (a) there were no main effects (i.e. terms (1) and (2) in the model above were all 0), and (b) there were no interaction effects (i.e. terms (3) in above model were all 0). These linear hypothesis tests were carried out using the “linearHypothesis” function in the “car” package [58] and with the anova function in R. Fisher’s method was used to combine these p-values from males and females [59]. Similar results were obtained using a full 3-way interaction model including all interactions between sex, maternal substitution and paternal substitution. In this approach, the test of the null hypothesis that all main effects in males and females were 0 had a p-value of 3.168e-05 and 1.17e-05 for weight and glucose respectively, while the overall test for interaction had a p-value of 0.44 and 0.00011 for weight and glucose respectively. Inverse-variance meta-analysis was used to combine the coefficient estimates from the males and females. If ${\hat{\beta }}_{m}$ and ${\hat{\beta }}_{f}$ are estimated genetic effects for males and females respectively then the IVW estimator is ${\hat{\beta }}_{IVW}=w{\hat{\beta }}_{f}+(1-w){\hat{\beta }}_{m}$ where $w=\frac{1/\mathrm{var}\left({\hat{\beta }}_{f}\right)}{1/\mathrm{var}\left({\hat{\beta }}_{f}\right)+1/\mathrm{var}\left({\hat{\beta }}_{m}\right)}$. Thus, while the genetic effects may potentially differ between males and females, the combined results represent a weighted average of the effect in males and in females. To account for potential non-normality, heteroscedasticity and *multiple testing*, we created 10,000 bootstrap data sets by sampling with replacement from each cross and sex combination. Studentized bootstraps (i.e. using pivotal statistics) were used to create confidence intervals for the coefficients and p-values. Multiple tests were adjusted for by comparing the observed test statistics to the maximum bootstrap test statistic as described elsewhere [60]. P-values were adjusted for multiple comparisons separately for each trait and separately for the main effects and interactions. As an alternative to the meta-analysis approach, we also fit a linear model adjusting for sex as a covariate. Results of this analysis are reported in S12 and S13 Tables. The proportion of the genetic variance explained by interactions was estimated as (R_Full_−R_Additive_)/ R_Full_ where R_Additive_ and R_Full_ are the adjusted coefficients of determination for the model with only main effects and for the full interaction model respectively. The adjusted coefficients of determination are an estimate of the proportion of variation in the trait which is explained by the model. Note that R_Full_ and R_Additive_ share the same denominator (i.e. the total trait variation). Thus, total trait variation cancels out of the quantity (R_Full_—R_Additive_)/ R_Full_ so that the quantity represents the amount of genetic variation that cannot be explained by main effects only. Using the adjusted version of the coefficient of determination helps account for potential overfitting. Bootstrap confidence intervals of this proportion were calculated.

### Sample preparation for RNA-Seq

Liver tissue stored in RNAlater was homogenized using a Tissumizer Homogenizer (Tekmar, Cincinnati, OH, USA). Total RNA was isolated using the PureLink RNA purification kit (Thermo Fisher Scientific, Waltham, MA, USA). A sequencing library was generated using the TruSeq Stranded Total RNA kit (Illumina, San Diego, CA, USA). RNA samples were sequenced on Illumina HiSeq2500s with single-end 50 base pair reads [61]. Library preparation and RNA sequencing were performed by the CWRU genomics core (Director, Dr. Alex Miron). A total of 7,269,450,186 reads were generated across four flow cells, with an average of 47,204,222 ± 928,913 [range: 14,561,990–76,538,825] reads per sample. Sequencing quality was assessed by FastQC [62], which identified an average per base quality score of 35.46.

### RNA-Seq data analysis

To maximize statistical power, 20 samples were selected for analysis from the control B6 group, 8 samples were selected from the single CSS groups, and 5 samples were selected from the double CSS groups. A total of 154 control and CSS mice were analyzed, including 20 B6 mice, 63 mice that were heterozygous for one A/J-derived chromosome, and 71 mice that were heterozygous for two different A/J-derived chromosomes. Only male mice were analyzed to avoid complications due to sex differences in gene expression. The B6.A4 x B6.A3 and B6.A8 x B6.A3 crosses were poor breeders and thus we did not obtain 5 samples to analyze from these crosses.

Reads were aligned using TopHat2 (2.0.10) [63] to the reference mm10 genome with the GENCODE vM7 annotations as a guide. Because the reference genome is comprised of sequence from strain B6, sequencing reads from a B6-derived chromosome are more accurately mapped than reads from an A/J-derived chromosome [64]. To avoid potential mapping biases, we created an “individualized genome” of the A/J mouse strain using the program Seqnature [64] with variant calls from the Mouse Genomes Project that were downloaded from The Sanger Institute [65]. Reads that were not mapped to the B6 genome were then mapped to the individualized AJ genome with TopHat2. HTSeq-count [66] and the GENCODE vM7 gene annotations[67] were used to count the number of reads for each gene feature. After filtering to remove duplicate reads, unmapped reads, low quality reads, and reads mapped to non-GENCODE regions of the genome, an average of 16,506,775 ± 439,754 [range: 4,638,701–30,465,477] reads were mapped to GENCODE regions per sample. There was no significant difference in the mapping efficiency (number of mapped reads / total number of reads) between the control B6 samples and any of the CSS strains either genome-wide (S10A Fig) or on the substituted chromosome (S10B Fig). This suggests that the sequence differences on the A/J chromosomes did not reduce mapping efficiency in the CSSs.

Graphical depictions of the distribution CPM (counts per million) were used to remove the following 3 outlier samples: E171, E305, and E570 (S1 Table). Genes where less than 75% of the samples had a count greater than or equal to 15 were considered to be expressed at low levels in liver and were removed leaving 13,289 genes that were considered expressed. To enhance reproducibility and reduce the dependence between the genes, svaseq [68] was used to create 5 surrogate variables that served as covariates in subsequent modeling.

EdgeR [69] was used to fit a model with main effects and pairwise interactions between each chromosome substitution. EdgeR uses a log link function, and thus departure from additivity in EdgeR is departure from a multiplicative model on the gene expression level. For each gene an interaction model was fit which included the following terms: (1) maternal substitution, (2) paternal substitution, (3) the interaction of maternal by paternal substitution, and (4) the SVA covariates. For all models, “B6” was used as the reference for the categorical chromosome substitution predictors.

A stratified FDR approach was used for the analysis of both meQTLs and ieQTLs [70]. For meQTLs, we tested for associations between every combination of chromosome substitutions in the study with every unfiltered gene in the RNA-Seq data. These hypothesis tests were stratified by chromosome and cis vs. trans. The method of Benjamini and Hochberg [71] was applied within each strata to control the false discovery rate. Similarly, the hypothesis tests for the ieQTLs were stratified by each chromosome combination and cis/trans. The stratified FDR approach has been shown to be more powerful when the proportion of true hypothesis differs by strata. The chromosome-chromosome interactions with FDR < 0.05 were divided into the categories synergistic and antagonistic based on the gene expression differences between the double CSS strain and the control strain relative to that predicted by an additive model (S9 Fig). Spearman’s r was used to summarize the association between several variables in the analysis. A Spearman’s r of 1 implies that the rank order of the values for two variables is the same. To estimate the amount of variation attributable to interaction, we fit an additive model in EdgeR which did not include any interaction terms. We then calculated for each individual and gene the fitted values assuming that the individual’s covariates (i.e. the SVA surrogate variables) were set to 0 and thus do not contribute to the variation. We calculate SS_Full_ as the sum of the mean centered and squared fitted values for the full model including interaction, S_Additive_ was calculated similarly for the additive model. We calculated the proportion of the genetic variation explained by interactions as (SS_Full_—S_Additive_) / S_Full_. This proportions is only meaningful when there is genetic variation to be explained. To filter out only genes with evidence of genetic control, using the full model for each gene, we tested the overall joint null hypothesis that all mouse strains had the same average expression level using the empirical Bayes quasi-likelihood F-tests test as implemented in EdgeR. This allowed us to classify some genes as showing evidence of genetic control. Only these genes were looked at further. The estimator (SS_Full_—S_Additive_) / S_Full_ may be slightly biased upward due to overfitting. However, the mean value for this statistic among the genes with no significant interaction (FDR > 0.5) was 0.25 (1^st^ quartile: 0.20, 3^rd^ quartile: 0.32) (Fig 5B), which gives one estimate of the upper bound on the possible bias. Here, the overall test that the interaction terms were all 0 was carried out using the Bayes quasi-likelihood F-tests test as implemented in EdgeR. To assess any potential bias stemming from the arbitrary selection of an FDR > 0.5, we performed a simulation study to independently approximate the upper limit on this bias. Using the fitted values (i.e. predicted mean) from the additive model described above, we simulated counts for each gene and individual from a Poisson distribution. The full and additive model was fit to the simulated data set, and the variance explained (SS_Full_—S_Additive_) / S_Full_ was calculated for each gene. The simulation was repeated 100 times and the average variance explained by interaction was averaged across all simulations for each gene. The mean for the amount of genetic variance explained by interaction under this simulated additive model was 0.13 (1^st^ quartile: 0.05, 3^rd^ quartile: 0.19) (Fig 5C). This gives another estimate of the upper bound on the possible bias.

### Multiple testing correction

For both the analysis of mouse phenotypes and RNA-Seq data it is necessary to account for multiple testing in order to avoid a large number of false positive findings. The approaches to multiple testing for the mouse phenotypes and RNA-Seq data are fundamentally different because the number hypotheses being tested were very different. For the mouse phenotype data, there were a relatively small number of targeted hypotheses, and thus the conservative and more confirmatory approach of controlling the family-wise type I error was applied. In this case, the genetic scan for each of the small number of traits was considered to be a separate question (i.e. the main effects for each trait and interaction effects for each trait were considered a separate “family” of hypotheses). For the large number traits analyzed in the RNA-Seq data, a less conservative and more hypothesis generating approach known as the stratified FDR was applied.

### Quantitative PCR (qPCR)

Tissue was homogenized using TissueLyser II (Qiagen, Valencia, CA, USA) and total RNA was isolated using the PureLink RNA purification kit with TRIzol protocol (Thermo Fisher Scientific, Waltham, MA, USA). Total RNA was reverse transcribed using the high capacity cDNA reverse transcription kit (Applied Biosystems, Carlsbad, CA, USA). The sequences for each primer are listed in S14 Table. The qPCR reactions were performed with the power SYBR green PCR Master Mix (Thermo Fisher Scientific, Waltham, MA, USA) and run on a Bio Rad CFX Connect Real Time System (Bio Rad, Hercules, CA, USA). Expression levels were calculated using the ΔΔCt method relative to the *Rplp0* control gene.

## Supporting information

S1 Fig Body weight and glucose levels in all CSS and control mice. Body weight and plasma glucose levels were measured in 5-week-old mice that were fasted overnight. Each dot represents the data from an individual mouse. Females (F) are shown in red. Males (M) are shown in blue. Outliers, as described in the Trait Analysis paragraph in the Methods section, are not shown but all data is available in S1 Table.
(EPS)

S2 Fig Schematic diagram of CSS and control crosses. Crosses were used to generate control, single CSS, and double CSS mice to examine main effects and interaction effects on various traits and gene expression levels. The four crosses used (top) to generate the control and CSS offspring (bottom) to study the substitution of chromosomes 3 and 10 are provided as an example of the crosses that were performed. Each rectangle represents a chromosome, with the substituted chromosomes 3 and 10 diagramed in this figure, on B6 background in all mice. The control B6 mice were generated from Cross I. The single CSS mice were generated from crosses II and III. The double CSS mice were generated from cross IV. M, Male. F, Female.
(EPS)

S3 Fig Inter-chromosomal epistasis regulates fasting glucose levels. Plots representing four out of the five CSS crosses that showed significant inter-chromosomal interactions on plasma glucose levels (The other significant CSS cross is shown in Fig 1). Each dot represents a single mouse. “Others” represents the data from all mice in this study excluding the 4 strains shown in that panel. The black horizontal line indicates the mean glucose level for each group. The red horizontal line indicates the predicted trait level based on a model of additivity.
(EPS)

S4 Fig Identification of 5 inter-chromosomal epistatic interactions that regulate fasting glucose levels in mice. Multiple testing adjusted p-values for interaction effects on fasting plasma glucose levels among 15 crosses each involving two A/J-derived chromosome substitutions with the substituted chromosomes indicated below the chart. Inverse-variance meta-analysis was used to combine the effects from males and females. The horizontal line indicates the significance threshold of 0.05.
(EPS)

S5 Fig Positive correlation between cis-meQTLs and trans-meQTLs. (A) Scatter plot of the relationship between the percentage of cis-meQTLs and trans-meQTLs in each of 8 CSS strains with one substituted chromosome. The strains are labelled on the graph with only their substituted chromosome, for example strain (B6 x B6.A8)F1 is shown for simplicity as A8. Data is shown on a log scale. (B) Histogram illustrating the percentage of cis-meQTLs and trans-meQTLs in each of 8 CSS strains with one substituted chromosome.
(EPS)

S6 Fig Identification of 5 trans-meQTLs that regulate the hepatic expression of Brca2. Gene expression levels of Brca2 in the liver are shown for strain B6 and 8 single CSS strains. Each dot represents Brca2 expression in an individual mouse. The mean value for each strain is indicated by a solid line. The Brca2 gene is located on mouse chromosome 5. ** indicates p<0.01 relative to strain B6. *** indicates p<0.001 relative to strain B6.
(EPS)

S7 Fig Regulation of hepatic Zkscan3 expression by additive meQTLs. (A) Gene expression of Zkscan3 in the liver was analyzed by (A) RNA-Seq and (B) RT-qPCR. Each dot represents Zkscan3 expression levels in an individual mouse. RT-qPCR data shown is relative to the control gene Rplp0. The mean value for each strain is indicated by a black line. The expected expression level of Zkscan3 based on a model of additivity is indicated with a red line. The p value from a test for interactions is shown. A p > 0.05 is suggestive of regulation by additivity rather than interactions.
(EPS)

S8 Fig Positive correlation between cis-ieQTLs and trans-ieQTLs. (A) Scatter plot of the relationship between the percentage of cis-ieQTLs and trans-ieQTLs identified among 15 pairwise CSS crosses. The data points are labelled on the graph with the two substituted chromosomes for each pairwise cross. Data is shown on a log scale. (B) Histogram illustrating the percentage of cis-ieQTLs and transieQTLs in each of 15 pairwise CSS crosses.
(EPS)

S9 Fig Schematic diagram illustrating the categorization of epistasis as either synergistic or antagonistic. Hypothetical mean expression levels are shown with black lines for the strains B6 and the two single CSS strains (CSSa x B6)F1 and (B6 x CSSb)F1, where a and b represent any two different substituted chromosomes. The predicted expression levels based on a model of additivity in the double CSS strain (CSSa x CSSb)F1 is shown with a red line. Synergistic epistasis is represented by a difference in trait values between the double CSS and control B6 strain that is greater than that predicted by additivity. Antagonistic epistasis is represented by a difference in trait values between the double CSS and control B6 strain that is less than that predicted by additivity. (A) Illustrates the case where only one single CSS strain shows expression differences relative to the control. (B) Illustrates the case where both single CSS strains show expression differences relative to the control. (C) Illustrates the case where both single CSS strains show expression differences relative to the control, but in opposite directions. (D) Illustrates the case where both neither single CSS strain show expression differences relative to the control.
(EPS)

S10 Fig No differences in mapping efficiency of RNA-Seq reads between B6 and CSSs. (A) Genome-wide mapping efficiency was calculated as the number of unique reads mapped to the GENCODE coding portion of the genome divided by the total number of reads per sample. (B) Mapping efficiency was calculated as above for the individual substituted chromosomes in each CSS as indicated.
(EPS)

S1 Table Summary of metabolic data for 766 mice used in analysis of body weight and plasma glucose. (XLSX)

S2 Table Number of mice used for analysis of body weight and plasma glucose. (XLSX)

S3 Table Main and average effects on phenotypes. (XLSX)

S4 Table List of meQTLs in CSSs. (XLSX)

S5 Table Main effects on gene expression. (XLSX)

S6 Table Summary of genes with multiple meQTLs. (XLSX)

S7 Table List of genes with multiple meQTLs. (XLSX)

S8 Table Genes examined by RNA-Seq and RT-qPCR for epistasis and additive interactions. (XLSX)

S9 Table List of ieQTLs in CSSs. (XLSX)

S10 Table List of genes with multiple ieQTLs. (XLSX)

S11 Table Summary of genes with multiple ieQTLs. (XLSX)

S12 Table Identification of fasting glucose QTLs using a combined linear model. (XLSX)

S13 Table Identification of body weight QTLs using a combined linear model. (XLSX)

S14 Table Primer sequences for RT-qPCR detection. (XLSX)
